# Content analysis of media coverage of breastfeeding in Mexico

**DOI:** 10.1111/mcn.12905

**Published:** 2019-12-15

**Authors:** Isabel Ferré‐Eguiluz, Gabriela Buccini, Amber Hromi‐Fiedler, Natalia Rovelo, Teresita González de Cosío, Juan Ricardo Pérez‐Escamilla‐Costas, Juan Ricardo Pérez‐Escamilla‐González, Rafael Pérez‐Escamilla

**Affiliations:** ^1^ Health Department Universidad Iberoamericana Mexico City Mexico; ^2^ Department of Social and Behavioral Sciences Yale School of Public Health New Haven Connecticut; ^3^ Eficiencia Informativa Mexico City Mexico

**Keywords:** breastfeeding, breastfeeding gear model, legislation, media coverage, Mexico, policy, SWOT analysis

## Abstract

Media can be a powerful communication tool to promote breastfeeding programs, influence mother's breastfeeding behaviour, and generate support among stakeholders for breastfeeding. Yet, there is little information on how media coverage influences a country's breastfeeding enabling environment. This study addressed this gap by conducting a retrospective content analysis of documents published between January 1, 2017 and January 1, 2018 to analyse the media coverage related to breastfeeding in Mexico. Content analysis was based on the breastfeeding gear model and a strategic planning technique to identify strengths, weaknesses, opportunities, and threats for enabling the national breastfeeding environment. Media coverage of breastfeeding was more frequent in August (36% of all documents). The top three topics commonly covered by the media were advocacy events promoting breastfeeding, promotion campaigns, and changes in breastfeeding legislation and policy. In general, the media coverage focused on strengths of specific breastfeeding policies. There was limited news coverage of key factors that negatively influenced or threatened the breastfeeding environment. Findings support the need to design strategies to engage the media covering in more depth and breadth diverse aspects of breastfeeding protection, promotion, and support efforts in Mexico.

**Key messages**

- Media coverage is needed to develop public interest and advocate for decision making in breastfeeding protection, promotion, and support.
- Breastfeeding media coverage in Mexico is primarily concentrated in the month of August due to World Breastfeeding Week. There is limited news coverage of key factors that negatively influence or threat the breastfeeding environment in Mexico.
- It is important to design and evaluate behaviour change social marketing campaigns in Mexico that include accurate and substantial media coverage.

## 1 INTRODUCTION

Breastfeeding is recognized globally as a human right for women and children (Kent, 2006; Perez‐Escamilla & Sellen, 2015; United Nations Human Rights, 2016) due to the indisputable evidence demonstrating that it is the most cost‐effective intervention to reduce infant mortality and promotes optimal maternal and infant health (Rollins et al., 2016; Victora et al., 2016). Long‐term breastfeeding can improve human capital and national development in low‐, middle‐, and high‐income countries by optimizing infant growth and cognitive development (Rollins et al., 2016; Victora et al., 2016). Investing in affordable and high‐quality breastfeeding programs, can help governments ensure that all mothers who wish to breastfeed are being supported. However, to invest, governments must be willing to consider the evidence and demonstrate commitment towards moving breastfeeding policies and programs forward.

Media can be a powerful communication tool to promote breastfeeding programs, influence mother's breastfeeding behaviour, and generate support among stakeholders for breastfeeding; the latter of which is key to the successful implementation of breastfeeding programs (Pérez‐Escamilla, Curry, Minhas, Taylor, & Bradley, 2012). Indeed, media coverage can influence policymakers' reactions to improving breastfeeding policies and programs within their countries, thus setting up the agenda for breastfeeding and ultimately enforcing the implementation and scaling up of breastfeeding programs (Buccini, Harding, Hromi‐Fiedler, & Pérez‐Escamilla, 2018). In fact, media helps to discover public interests and legitimize them as a problem, given its influence in determining which political issues are most important and urgent to solve, hence establishing political priorities in the public agenda (Bou‐Karroum et al., 2017; McCombs & Reynolds, 2002; Olper & Swinnen, 2009).

There is little information on how media coverage on breastfeeding influences a country's breastfeeding enabling environment (Bridges, Howell, & Schimied, 2018; Brown & Peuchaud, 2008; DeMarchis, Ritter, Otten, & Johnson, 2018). This study addressed this gap by conducting a review to document the degree and content of media coverage related to breastfeeding in Mexico following the assessment of the country's breastfeeding enabling environment in 2016 (González de Cosío, Ferré, Mazariegos & Pérez‐Escamilla, 2018).

Mexico was chosen because it has one of the lowest rates of exclusive breastfeeding in Latin America and the Caribbean; between 2006 and 2012, exclusive breastfeeding decreased from 22.3% to 14.4%, subsequently increasing only slightly to 16% in 2016 (González de Cosío, Escobar‐Zaragoza, González‐Castell, & Rivera‐Dommarco, 2013). This decline was especially pronounced in rural areas and other socioeconomically vulnerable groups (González de Cosío et al., 2013). In response, in 2016, Mexico implemented the Becoming Breastfeeding Friendly (BBF) initiative (Pérez‐Escamilla et al., 2018) to assess the country's readiness and capacity for scaling up effective breastfeeding programs through a highly intersectoral and participatory process (González de Cosío, Ferré, Mazariegos & Pérez‐Escamilla, 2018). The BBF experts committee identified several points that potentially explain why Mexico has low breastfeeding rates. First, the country lacks a mechanism for monitoring and enforcing the World Health Organization Code of Marketing of Breastmilk Substitutes. Second, the maternity leave for working women in the formal sector is insufficient (12 weeks) and inexistent for those working in the informal economy. Third, there is no national budget to promote, protect, and support breastfeeding. Fourth, health providers training does not meet the minimum breastfeeding/human lactation education curriculum standards recommended by World Health Organization. Lastly, only 11% of the hospitals have the Baby‐friendly Hospital Initiative certification (González de Cosío, Ferré, Mazariegos, & Pérez‐Escamilla, 2018). The BBF assessment resulted in the development of recommendations tailored to the country's needs as well as wide spread dissemination to key stakeholders and decision makers (González de Cosío, Ferrét, Mazariegos, & Pérez‐Escamilla, 2018). Although these activities were intended to improve the scaling up of the breastfeeding environment within the country, little is known about the upstream factors that support effective implementation of these recommendations.

## 2 METHODS

### 2.1 Design

A retrospective content analysis of media documents was conducted using print and online media sources in Mexico between January 1, 2017 and January 1, 2018. In this paper, media documents were considered any newspaper coverage (including journal or magazine articles, reports, and editorials) and radio coverage (including audio of radio news, programs, and interviews).

### 2.2 Setting

Mexico is an upper–middle income country located in the Latin American and Caribbean region (World Bank, 2019). In 2015, the estimated population was 119,938,473 people with 2,293,708 births per year (Instituto Nacional de Estadística y Geografía, 2018a,b). A public perceptions survey found that the top media channels used by the Mexican population to keep informed on science and technology were magazines (48.7%) and newspapers (43.8%) followed by television (26.6%) and radio (9.7%; Instituto Nacional de Estadística y Geografía, 2018a,b).

### 2.3 Media review

An online search of media documents from newspapers and radio platforms was conducted using a combination of the following key words in Spanish: breastfeeding, maternity leave, Becoming Breastfeeding Friendly, Permanent Interinstitutional Support Group for Breastfeeding (GILPALM for its acronym in Spanish). The media search was conducted pro bono by the company Eficiencia Informativa (https://www.efinf.com/) that specializes in media monitoring and content analysis for diverse stakeholders including government, decision makers, and corporations. The media search was designed to meet the following inclusion criteria: (a) published within the timeframe of January 1, 2017 to January 1, 2018; (b) related to breastfeeding in Mexico; and (c) not an advertisement.

**Figure 1:**
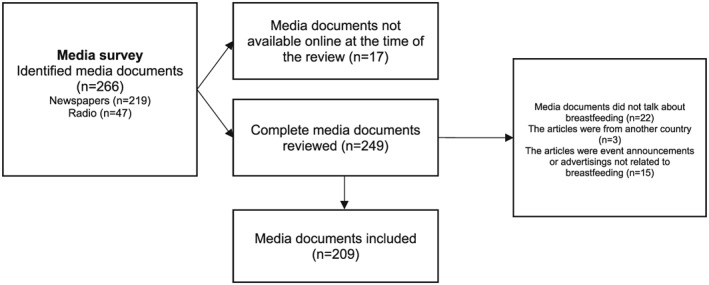
Flowchart with steps followed to identify breastfeeding media coverage in Mexico

Following the identification of 266 links of media documents from the initial search, two researchers (IF and NR) reviewed them to confirm that they met the inclusion criteria. Of these, 40 media documents did not meet the inclusion criteria and 17 media documents were not available online by the time of the review. A total of 209 media documents were included in the final sample, of which 175 correspond to newspapers and 34 to radio (Figure 1). The included media documents were then organized by media platform (newspaper or radio). The media documents were sorted by date to summarize the frequency of media mentions by month.

### 2.4 Data analysis

The media content analysis was conducted in two steps:

1. *Classification of media documents into themes*. First, media documents were classified into themes on the basis of the breastfeeding gear model (BFGM) domains (Pérez‐Escamilla et al., 2012). The BFGM is a conceptual framework that stipulates that eight gears—Advocacy; Political Will; Legislation and Policies; Funding and Resources; Training and Program Delivery; Promotion; Research and Evaluation; and Coordination, Goals, and Monitoring—must work in harmony for scaling up effective national breastfeeding programs (Pérez‐Escamilla et al., 2012). The BFGM is based on the premise that evidence‐based advocacy generates political will to enact policies and legislation necessary to protect, promote, and support optimal breastfeeding practices. These, in turn, are needed to generate the necessary resources to train the workforce and delivery of programs, as well as to promote breastfeeding through behaviour change communication campaigns. Implementation evaluations are necessary to maintain the quality and success of the programs. Finally, a master gear is needed to provide intersectoral coordination and goals' monitoring to ensure high coverage and quality of breastfeeding policies and programmes. This gear also facilitates the flow of information across the rest of the gears, providing timely feedback on the actions needed to improve or maintain the high quality of scaled up programs (Pérez‐Escamilla et al., 2012). We developed a code structure on the basis of the BFGM to guide the classification of media documents into themes and subthemes. Each gear of the BFGM was considered a theme following an operational definition (Table 1).
  Media documents were coded independently by two researchers standardized against each other (IF and NR), following the coding structure. Each researcher read and then labelled each media document with at least one theme on the basis of the main message(s). Any differences in the researchers coding of the main themes of the media documented were resolved through a consensus process and, if necessary, by consulting a third researcher (GB with expertise in breastfeeding. The frequency of each theme, as well as subthemes generated during this process were summarized. Of the 209 media documents included, 77 were classified into two or more themes.
2. *Classification of themes*. Themes were classified using a strategic planning technique to identify strengths, weaknesses, opportunities, and threats (SWOT) for enabling the national breastfeeding environment. SWOT analysis consists of an examination of an organization's or an environment's internal strengths and weaknesses, its opportunities for growth and improvement, and the external threats present to its survival. It is a decision‐making tool that sets the stage for identifying strategic options to maximize organizational or environmental performance (van Wijngaarden, Scholten, & van Wijk, 2012). Originally, SWOT was designed for use by industries and business, however, recently, it has been applied in the healthcare contexts (Harrison, 2010).
  We analysed and classified the themes identified in Step 1 into the SWOT matrix. The analysis was conducted independently by two researchers standardized against each (IF and NR) following the SWOT operational definitions (Table 2). A third researcher (GB) was consulted when consensus in the classification was not reached. This classification generated a SWOT matrix of media coverage for enabling breastfeeding environment in Mexico.

**Table 1:** Coding structure according to the breastfeeding gear model (Perez‐Escamilla et al., 2012) and the Becoming Breastfeeding Friendly Index (Pérez‐Escamilla et al. 2018). http://www.bbf.yale.edu

Themes	Definition
Advocacy gear	It is the effort for translating evidence‐based recommendations into actions to promote breastfeeding. Advocacy seeks through a massive social mobilization engage people and resources to generate enough political pressure to influence political will
Political will gear	It is the expressed, institutional, and budgetary commitment on the part of a government to carry through a policy. Political will exists when a sufficient set of decision makers with a common understanding of a particular problem on the formal agenda is committed to supporting a commonly perceived, potentially effective policy solution
Legislation gear	It is the establishment and enactment of national laws, norms, regulations, and policies on breastfeeding that demonstrate a national commitment to scale‐up, promote, and support breastfeeding programs and initiatives
Funding and resources gear	It is the budget of a government for a specific activity. National funding strategies that demonstrate a national commitment to scale‐up breastfeeding programs, for example, (a) specific pay line for funding breastfeeding policies and programs and (b) provide a formal mechanism to fund maternity entitlements
Training and program delivery gear	Training: It is the training provided to health care providers about attitudes, knowledge, and skills on breastfeeding counselling and lactation management. Program delivery: It is the activities planned and delivery at all levels of health care, including facility‐based programs (such as the baby‐friendly hospital initiative) and community‐based programs (including mother‐to‐mother support activities)
Promotion gear	It is the use of a variety of methods (including social media, national and local events, campaigns, community activities, and interpersonal skills) to convey breastfeeding messages to targeted audiences
Research and evaluation gear	It is a sound multilevel monitoring and evaluation system is needed to ensure that the breastfeeding programs are being properly implemented and to share information from the local to the national level and to enable proper decision making, at each level, in a timely fashion.
Coordination, goals and monitoring gear	The synchronization and integration of activities, responsibilities, and structures of command and control to ensure that government resources are used in the most efficient way to adequately fulfill the function of breastfeeding policy.

Abbreviation: BFHI, baby‐friendly hospital initiative.

**Table 2:** Operational definitions for strengths, weaknesses, opportunities, and threats (SWOT) analysis

Status	Positive	Negative
Currently implemented	Strengths Are those situations or messages that are being implemented currently or that can be controlled directly and that positively affect the enabling of the breastfeeding friendly scale‐up environment	Weaknesses Are those situations or messages that are being implemented currently or that can also be directly controlled and that negatively affect the enabling of the breastfeeding friendly scale‐up environment
	Example: Training strategies for health professionals on breastfeeding issues	Example: Insufficient budget for the operation of the policy of breastfeeding
Not currently implemented	Opportunities Are those situations or messages that are not being implemented currently or that are not controllable and that positively affect the enabling of the breastfeeding friendly scale‐up environment	Threats Are external factors or challenges or messages that are not being implemented currently or that are not controllable and that negatively affect the enabling of the breastfeeding friendly scale‐up environment
	Example: Extension of the duration of maternity leave	Example: Possible cancellation of the application of the National Health Survey that collects information on breastfeeding

## 3 RESULTS

**Figure 2:**
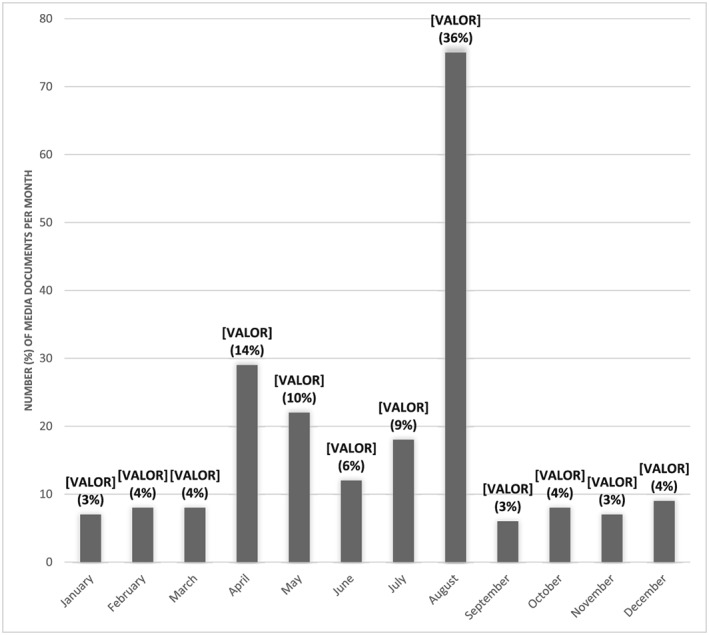
Number (percentage) of media documents published per month in 2017

Breastfeeding was covered in the media every month, with August having the highest frequency of breastfeeding media documents published (36%), corresponding to the month when World Breastfeeding Week was launched (Figure 2). April was the second month with highest frequency of media documents published (14%), followed by May (10%), and July (9%).

The themes within the BFGM framework that emerged most frequently in media documents were related to advocacy (*n* = 77, 36.8%), breastfeeding promotion (*n* = 73, 34.9%), and legislation and policy (*n* = 71, 33.9%; Table 3). However, very few media sources were related to two themes: funding and resources (*n* = 7, 3.3%) and political will (*n* = 1, 0.4%). Furthermore, no media documents were related to the breastfeeding national coordination theme. Within the advocacy theme, World Breastfeeding Week garnered the most media attention. The implementation of work site lactation rooms garnered the most media coverage within the legislation and policy theme. With regards to breastfeeding promotion, media coverage focused on the benefits of breastfeeding. Concerning training and program delivery, the topics more extensively covered were the implementation of the Baby‐friendly Hospital Initiative, human milk banks, and breastfeeding counselling. Lastly, the topic that received more attention for research and evaluation focused on breastfeeding rates in Mexico.

**Table 3:** Number of breastfeeding documents by themes and subthemes according to the breastfeeding gear model reported by the media in Mexico in 2017

Themes	Media documents	Subthemes	Media documents
		World breastfeeding week	57
		Early childhood pact	14
		Becoming breastfeeding friendly index	3
		Forum towards the law of breastfeeding	1
Advocacy	77	Call to action to protect breastfeeding during the earthquake	1
		Recommendation from the National Human Rights Commission for the Mexican Social Security Institute guarantee the enjoyment of the full licence of maternity in premature delivery	1
Political will	1	The political will of health officials is insufficient	1
		Implementation of breastfeeding rooms	39
		State laws for the protection and promotion of breastfeeding	11
Legislation and policies	71	International code of marketing of breast‐milk substitutes (code)	7
		National breastfeeding strategy	1
		Practical guide “breastfeeding in the workplace”	1
Funding and resources	7	Insufficiency budget to implement actions of protection, promotion and support to breastfeeding	3
		The costs that inadequate breastfeeding practices generate for the country	4
Training and program delivery	56	The implementation of programs such as baby‐friendly hospital initiative, milk banks, and breastfeeding counselling	44
		Health providers in‐service training	8
		Health providers preservice training	3
Promotion	73	Benefits of breastfeeding	58
		Promotion strategies that are being implemented	23
Research and evaluation	44	Breastfeeding rates in Mexico	42
		Nationals surveys that collect information on breastfeeding practices	2
Coordination, goals, and monitoring	0		

The analyses also showed that both strengths (i.e., positive aspects that are currently in implementation in the country) and opportunities (i.e., positive aspects that are not currently in implementation in the country) were the SWOT dimensions most frequently addressed in the media (Table 4). The media content analysis showed that there was very limited news coverage of key factors that negatively influence the breastfeeding environment including: (a) lack of political will of health officials; (b) infractions of the World Health Organization Code of Marketing of Breastmilk Substitutes (The Code) by industry; (c) the inability of the government to generate funding for enabling the breastfeeding environment; and (d) breastfeeding campaigns designed and ran by companies that produce breast milk substitutes. The only news coverage addressing a threat to the breastfeeding environment was related to the possible cancellation of the national survey that collects data on breastfeeding practices.

**Table 4:** Strengths, weaknesses, opportunities, and threats (SWOT) analysis of breastfeeding subthemes covered in the media in Mexico in 2017

Status	Positive	Negative
Currently implemented	Panel A. strengths: Events in favour of breastfeeding as world breastfeeding week, early childhood pact, and becoming breastfeeding friendly index. Implementation of breastfeeding rooms Laws initiatives for the protection and promotion of breastfeeding Changes from weeks of pregnancy leave to postpartum National breastfeeding strategy Practical guide “breastfeeding in the workplace” Guarantee of the enjoyment of full maternity leave in case of premature birth. Health providers in‐service training The implementation of programs such as baby‐friendly hospital initiative, milk banks, and breastfeeding counselling The diffusion of breastfeeding benefits Promotion strategies that are being implemented Increase in rates of breastfeeding in Mexico	Panel B. weaknesses: The insufficient political will of health officials Leaving the monitoring of the code in the hands of the industry Insufficient budget for the protection and promotion of breastfeeding Breastfeeding campaign elaborated by companies that produce breast milk substitutes
Not currently implemented	Panel C. opportunities: Increase breastfeeding rates in Mexico The savings that would be generated from complying with the recommended breastfeeding practices Monitor and enforce the code Implement more breastfeeding rooms in workplaces Extend maternity leave Increase the budget for the protection and promotion of breastfeeding Health providers preservice and in‐service training Breastfeeding counselling	Panel D. threats: The possible cancellation of the national survey that collects information on breastfeeding practices in the country

## 4 DISCUSSION

Our study found that media coverage in Mexico focused mainly on breastfeeding events, breastfeeding rates and benefits, as well as implementation of worksite lactation rooms. Increasing the coverage of breastfeeding events and marketing its benefits in the mass media is desirable as this approach is a key component of effective behaviour change communication interventions (Menon et al., 2016). Moving forward, it is also important for mass media to report obstacles that women face to breastfeed as well as strategies used to cope with these challenges. This is necessary for decision makers and society at large to understand the comprehensive support that women need to make breastfeeding work. Lactation rooms in the place of employment were identified as a top priority by the 2016 Mexico BBF committee, and this recommendation was strongly endorsed by decision makers (González de Cosío, Ferré, Mazariegos & Pérez‐Escamilla, 2018). This is encouraging as lactation rooms represent a relatively low‐cost intervention that has been associated with a reduction in employee absenteeism, improved workforce performance, commitment, and retention as well as improved breastfeeding at 6 months (Rollins et al., 2016). In this context, our finding that over half of the media documents (i.e., 34 articles) within the legislation and policy theme were related to this topic suggests strong media attention to fulfilling this commitment. This is an example of how media coverage has the ability to affect public opinion via people's attitudes, perceptions, beliefs, and behaviours (Otten, 1992), including influencing policymakers' reactions and decisions (Buccini et al., 2018). Thus, it is critical to use media's power more effectively in Mexico to help generate the political will to reinforce commitments towards strengthening the breastfeeding friendly environment.

Our findings showed important breastfeeding themes that were not addressed by the Mexican media. Media coverage on funding and resources and political will was limited, but there was no coverage on the country's coordination for breastfeeding programs. Coordination for breastfeeding is the master gear that drives the rest of the seven gears within the BFGM to promote a breastfeeding friendly environment (Pérez‐Escamilla et al., 2012). Without media support, advocating for improving coordination of breastfeeding programs and policies can be more challenging. Media can influence how people think about a topic (breastfeeding, for instance), what are the causes, how important it is, and possible solutions (Buckton et al., 2018). Therefore, framing these issues in the media could shape how the population and in turn decision makers think about this problem and what type of alternatives could be considered viable (Liu, Cai, & Zhao, 2018). For this reason, it is important to improve breastfeeding media coverage in Mexico perhaps by forming an alliance with journalists to position breastfeeding as a top public health issue on the national agenda as it happened in the case of the soda tax in Mexico (Vilar‐Compte, 2018).

Breastfeeding media coverage in Mexico was primarily concentrated in the month of August due to World Breastfeeding Week, which mobilizes national and international media attention towards the importance of breastfeeding. Evidence shows that just providing coverage during World Breastfeeding Week is not enough as when a topic is included in the media more frequently that specific issue has a greater chance of influencing public opinion (McCombs, Shaw, & Weaver, 2014). Hence, the lack of consistent coverage of breastfeeding topics throughout the year may not provide the dosage of exposure needed to shape public opinion and influence decision makers who have many competing demands (Liu et al., 2018). Given that the media determines the type, extent, and form of information that is published (Dorfman & Krasnow, 2014; McCombs, 2005), it is key for breastfeeding advocates in Mexico to develop better media coverage strategies.

In examining the strengths, opportunities, weaknesses, and threats (SWOT) of media content, one notable weakness was the lack of attention paid to the need for enforcement of the WHO Code (Hernández‐Cordero et al., 2018). In Mexico, breast milk substitute companies are expected to self‐regulate their compliance with the Code, however, self‐regulation of the industry has been shown to be ineffective (Kent, 2015; Sharma, Teret, & Brownell, 2010; Robinson, Buccini, Curry, & Pérez‐Escamilla, 2019). In this context, the media could become a powerful tool to report violations. Indeed, media platforms have been identified as an effective space for the general public and the stakeholders to engage with a specific topic specifically to make their voices heard (Gal‐Tzur, Grant‐Muller, Minkov, & Nocera, 2014). Therefore, policy debates through the media can help democratize policies as well as increase the awareness on breastfeeding, ultimately strengthening the advocacy and the demand for better breastfeeding programs.

Our study has some limitations to be considered. First, we could not examine breastfeeding‐related television coverage. Although originally included in the search plan, by the time the analyses were conducted, the links were no longer functional. Second, the search was restricted within a period of a year, which might limit the generalization of our findings. This timeframe was selected because we were interested in the short term of the media response to the BBF assessment presented early 2017. On the other hand, a particular strength of our study was that we used a systematic process to analyse the data using a previously validated framework BFGM (Pérez‐Escamilla et al., 2012) and SWOT analysis (Harrison, 2010).

To our knowledge, this is the first study specifically designed to understand breastfeeding media coverage related to breastfeeding in Mexico. Findings support the need to design strategies to engage journalists and the media much more in covering diverse aspects of breastfeeding protection, promotion, and support efforts in Mexico.

## ETHICAL STATEMENT

This study was exempt from Human Subjects Approval by the Yale University Institutional Review Board because it did not involve human subjects' research.

## CONFLICTS OF INTEREST

The authors declare that they have no conflicts of interest

## CONTRIBUTIONS

GB, AHF, and RPE designed the research protocol. IF, GB, and NR conducted the research and analysed the data. JRPEC and JRPEG coordinated the media searches. IF and GB wrote the first draft of the manuscript and AHF, RPE, TGC, JRPEC, and JRPEG revised it critically through an iterative process. All authors approved the final version of the manuscript.
